# Biomarkers for dietary fatty acid densities among postmenopausal United States females derived using a habitual-diet human feeding study

**DOI:** 10.1016/j.ajcnut.2026.101197

**Published:** 2026-01-14

**Authors:** Ross L Prentice, Lesley F Tinker, Marian L Neuhouser, Johanna W Lampe, Daniel Raftery, GA Nagana Gowda, Xiaoling Song, Sandi L Navarro, Ying Huang, Sowmya Vasan, Tonya S Orchard, Theodore M Brasky, JoAnn E Manson, Cheng Zheng

**Affiliations:** 1 Division of Public Health Sciences, Fred Hutchinson Cancer Center, Seattle, WA; 2 Departments of Biostatistics and Epidemiology, School of Public Health, University of Washington, Seattle, WA; 3 Department of Anesthesiology and Pain Medicine, University of Washington, Seattle WA; 4 Clinical Research Division, Immunotherapy Integrated Research Center, Fred Hutchinson Cancer Center, Seattle, WA; 5 Department of Human Sciences, Ohio State University, Columbus OH; 6 Department of Internal Medicine, Ohio State University, Columbus OH; 7 Department of Medicine, Brigham and Women’s Hospital, Harvard Medical School, Boston MA; 8 Department of Biostatistics, University of Nebraska Medical Center, Omaha NE

**Keywords:** dietary biomarker, fatty acid density, phospholipid fatty acid, serum metabolomics, urine metabolomics

## Abstract

**Background:**

Although measures of blood and tissue fatty acid (FA) concentrations are available, objective measures of dietary FA densities (grams per kilocalories) are generally lacking.

**Objectives:**

We aimed to explore the development of biomarkers for specific and composite dietary FA densities, not including contributions from dietary supplements, using metabolite profiles from serum and 24-h urine, along with separately measured serum phospholipid FA concentrations in the Women’s Health Initiative.

**Methods:**

Potential biomarker equations were based on linear regression of feeding study dietary FA densities on metabolite concentrations, each log-transformed, among participants in a habitual-diet human feeding study (*n* = 153) within the Women’s Health Initiative. Corresponding biomarker equations were also considered for total SFA, MUFA, and PUFA densities and for total n–3 and n–6 PUFA densities. Dietary FA density estimates derived from these equations were evaluated by correlation with feeding study intake densities, and by other important biomarker criteria.

**Results:**

Regression cross-validated *R*^2^ values >30% for specific SFAs were 64.7 butyric, 60.9 caprioc, 48.7 caprylic, 53.0 capric, 39.9 lauric, 61.0 myristic, 42.2 palmitic, 34.2 stearic, 34.8 arachidic, 49.9 decosanoic; for specific MUFAs were 31.3 oleic; and for specific PUFAs were 51.7 linoleic, 50.1 α-linolenic, 39.7 arachidonic, 40.2 EPA, 53.5 decosapentaenoic acid, and 47.9 DHA. Corresponding values were 46.4, 52.8, 46.1, and 52.4 for total SFA, total PUFA, total n–3, and total n–6 densities. Many FA density equations had contributions from multiple metabolites, mostly serum metabolites, and from total energy expenditure. Sensitivity and specificity criteria are plausibly satisfied for proposed biomarkers, based on the feeding study design and on the sets of selected metabolites.

**Conclusions:**

Combinations of log-transformed metabolite concentrations can lead to objective intake density estimates for multiple FAs in the diets of United States postmenopausal females, with relevance to the reliable study of dietary FA densities and chronic disease risk.

This study was registered at clinicaltrials.gov as NCT00000611 https://clinicaltrials.gov/study/NCT00000611).

## Introduction

The association of dietary fatty acid (FA) densities (grams of macronutrient per kilocalorie of total energy intake) with disease risk has been a major topic in nutritional epidemiology since a positive coronary artery disease risk association with SFA density and inverse associations with MUFA and PUFA densities, based on self-reported dietary data, were identified among US Nurses [1,2]. Subsequently, meta-analyses of cohort studies that also relied on self-reported dietary data have been variably supportive of any important role for these FA categories in relation to coronary artery disease, other cardiovascular diseases, or type 2 diabetes [[3], [4], [5], [6]]. Rather than study associations with self-reported dietary data, which may incorporate systematic measurement error, 1 large epidemiology cohort consortium group conducted large-scale studies of measured serum FA concentrations for association with clinical outcome risk [7]. However, as these investigators acknowledge, the FA densities studied assess FA dietary intake followed by metabolism rather than intake itself, and serum FA densities may not correlate strongly with dietary FA densities because of complex metabolism. Our goal in considering the development of objective biomarkers for dietary FA densities here is to yield intake measures that are free from systematic bias. Study of the systematic bias that may attend self-reported dietary composition assessments has been limited by the typical lack of objective intake measures for comparison.

Our Women’s Health Initiative (WHI) research group has recently proposed novel biomarkers for dietary macronutrient densities and for certain of their components [[8], [9], [10], [11], [12]], primarily based on serum and 24-h urine metabolomics profiles in the WHI Nutrition and Physical Activity Assessment study-Feeding study (NPAAS-FS). Proposed metabolomics-based biomarkers [12] for total SFA, MUFA, and PUFA densities had cross-validated percent of intake variation explained (CV-R^2^) >30% in the NPAAS-FS cohort [13]. The ability to identify biomarkers satisfying correlational criterion for these densities [12] but not for total fat density [14] suggests that even stronger intake biomarkers for specific dietary FA densities may be able to be developed using the same methodology. To aid in this exploration, we also have available separately measured relative serum phospholipid fatty acid (PLFA) concentrations [15]. The PLFA measures allow unsaturated FA double bonds to be classified as *cis* or *trans* providing an additional data dimension for dietary FA biomarker development.

Regression CV-*R*^2^ values serve as our primary quantitative outcome measure in the biomarker development analyses reported here, with sensitivity, specificity, and other biomarker evaluation criteria also considered.

## Methods

The context, resources, and methods for the dietary FA density biomarker development analyses reported here are similar to those for our earlier macronutrient density biomarker reports [[8], [9], [10], [11], [12]].

### Study cohorts

Briefly, during 1993–1998, 48,835 participants were randomized in the WHI dietary modification (DM) trial of a low-fat dietary pattern, and 93,676 participants were enrolled in the companion prospective WHI Observational Study (OS) [16]. All participants were postmenopausal and in the age range 50 to 79 y when enrolled at 40 US clinical centers. The WHI food frequency questionnaire (FFQ) [17] targeted dietary intake over the preceding 3-mo period and was administered at baseline and year 1, and approximately every 3 y thereafter in the DM trial throughout the trial intervention period (ended 31 March, 2005), and was administered at baseline and at year 3 in the OS. Nutrient intake estimates were derived using the University of Minnesota’s Nutrition Data System for Research (NDSR version 2005). Participants completed core questionnaires at WHI enrollment including medical history, reproductive history, family history, personal habits, medications and dietary supplements, and provided a fasting blood sample [16].

### WHI nutrition biomarker studies

We conducted an initial Nutrition Biomarker study among 544 participants in the DM trial cohort during 2004–2006 to study measurement properties of the WHI FFQ in this trial context [18]. That was followed by the NPAAS study among 450 OS participants during 2007–2009, conducted in part to study measurement properties of FFQs, 4-d food records and three 24-h dietary recalls [19]. The protocol for these studies required 2 clinic visits separated by 2 wk and included various at-home activities, including 24-h urine collection. A 20% reliability subsample repeated the respective protocols about 6 mo after their initial biomarker study participation. The first NPAAS visit included measured height and weight, doubly-labeled water (DLW) dosing for total energy expenditure (TEE) assessment [20], completion of FFQ, dietary supplement, and other questionnaires, and collection of a blood specimen. Participants received instructions and a kit for 24-h urine collection for home completion. At the second clinic visit, participants brought 24-h urine specimens collected over the preceding day, provided a fasting blood specimen, and provided additional spot urine specimens to complete the DLW protocol.

### NPAAS feeding study

We conducted the NPAAS-FS [13] among 153 WHI participants aged ≤80 y in the Seattle area from 2010 to 2014. These participants were enrolled in the DM trial comparison group (*n* = 29,294) or the OS. The Fred Hutchinson Cancer Center’s Human Nutrition Laboratory provided participants with food and some beverages over a 2-wk feeding period, with individualized diets that aimed to approximate participants’ usual diets, so that blood and urine concentrations would stabilize quickly and population intake variations would largely be retained in the feeding study cohort. Participants were permitted to continue their usual dietary supplement use during the feeding period, and each participant’s dietary supplement use was reported on food records.

**FIGURE 1:**
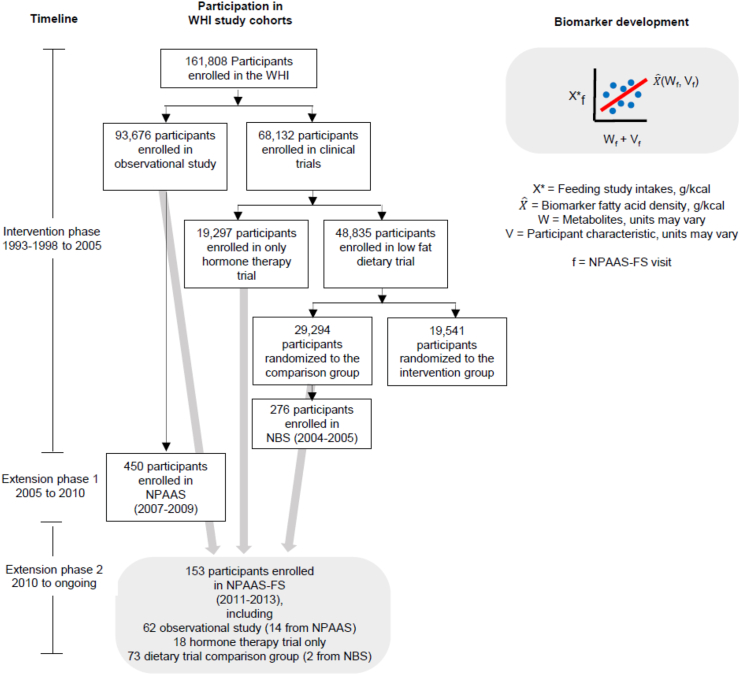
Participant flow in Women’s Health Initiative cohorts (1993–present), including NPAAS, NPAAS-FS, and NBS, with right side display of biomarker development. Details are provided in the narrative. NBS, Nutrition Biomarker study; NPAAS, Nutrition and Physical Activity Assessment study; NPAAS-FS, NPAAS feeding study; WHI, Women’s Health Initiative.

Aside from the ≤80 y age restriction in NPAAS-FS, included in part to enhance similarity of age groups across biomarker studies, the inclusion/exclusion criteria were essentially the same for the 3 nutrition biomarker studies. For NPAAS-FS enrollees in the OS, the DM trial comparison group, and the hormone therapy trial but not DM trial subsets of the WHI Seattle Clinical Center cohort were sent invitation letters, with initial invitations to those living within 50 km of the Fred Hutchinson Cancer Center for logistical reasons. From 450 invitations, 153 proved to be willing and not to be excluded by eligibility or exclusionary criteria, thereby reaching the targeted 150 sample size. This sample size was determined by statistical power considerations for studies of potential vitamin intake biomarkers based on serum vitamins, and potential dietary fatty acids intake biomarkers based on serum PLFAs. Potential participants were excluded if unable to satisfy criteria for the DLW energy expenditure protocol. if they reported trying to gain or lose weight, reported gaining or losing weight unintentionally, or experienced a very large (6.8 kg or larger) change in body weight over the preceding 4 wk. Participants having medical conditions that may interfere with their ability to complete protocol and data study requirements (e.g., kidney dialysis, diabetes), were also excluded [13]. Figure 1 shows participant flow in WHI cohorts leading to the NPAAS-FS enrollment from each of its 3 sources.

Participants in these biomarker studies had TEE and weight variation over the 2-wk DLW protocol period measured, as well as urinary nitrogen (UN) from 24-h urine samples as a biomarker of total protein intake [21]. Also measured were weight at the time of TEE assessment and at their preceding WHI enrollment about 10 y (median) earlier as well as waist circumference on each occasion. A percent body fat assessment from total body water [22] as measured in the DLW procedure, was available at the time of DLW assessment. Self-reported recreational physical activity (PA) was assessed by a WHI Personal Habits Questionnaire [23]. Biomarkers developed for the dietary intake densities studied here utilize metabolomics profiles from the second clinic visit serum and 24-h urine specimens, along with the potential inclusion of readily available participant characteristic measures.

Baseline demographic and lifestyle characteristics for participants in the NPAAS-FS cohort have been reported [13] and are replicated here in Supplementary Table 1. Briefly, participants were well educated (83% college degree or higher), and mostly nonsmokers (98%). Most self-identified as White (95%), had overweight or obesity (60%), and as a group were of similar ages to other WHI enrollees.

### Metabolite profiling

Serum and 24-h urine metabolomics profiles, obtained using specimens collected at the end of the NPAAS-FS feeding period, were derived in the Northwest Metabolomics Research Center (Daniel Raftery, Director) at the University of Washington [8,14], and serum PLFA concentrations were measured in the Public Health Sciences Biomarker Core Lab at the Fred Hutchinson Cancer Center (Xiaoling Song, Director).

#### Serum metabolite profiles

Briefly, serum samples from NPAAS-FS participants were analyzed by targeted liquid chromatography-mass spectrometry (LC-MS/MS) using liquid chromatography coupled to a Sciex Triple Quad 6500+ mass spectrometer. A total of 303 metabolites were targeted, of which 155 were detected with <20% missing values. Separately, lipid metabolites were measured using the Sciex QTRAP 5500 Lipidyzer platform, including the SelexION differential mobility spectrometry method that targeted 1070 lipids in 13 major lipid classes, resulting in 664 serum lipids that had <20% missing values.

#### Urine metabolite profiles

Metabolite profiles from 24-h urine samples were analyzed by nuclear magnetic resonance (NMR) spectroscopy using a Bruker Avance III 800 MHz NMR spectrometer. Relative concentrations for 57 targeted metabolites were obtained. None of the metabolites had >20% missing values. Urine metabolites were also analyzed by untargeted gas chromatography-mass spectrometry (GC-MS) method using an Agilent 7890A / 5875C instrument, resulting in the identification of 275 metabolites with <20% missing values. The full list of metabolites meeting quality criteria (including <20% missingness) using each of these metabolomics platforms is given in Supplementary Table 2 of [24]. Undetectable metabolite concentrations were imputed as one-half the smallest observed concentration.

For LC-MS/MS data, 17 metabolites had some imputed values, and for these variables, a mean of 6% of values were imputed. The corresponding numbers for lipidomics were 220 metabolites, with a mean of 5% of their values imputed; for NMR were 4 metabolites with a mean of 3% of values imputed; and for GC-MS were 36 metabolites with a mean of 3% of their values imputed. Median coefficients of variation from blinded pooled study QC samples were 4.4% for NMR, 5.6% for lipidomics, 7.2% for targeted LC-MS/MS, and 21.6% for GC-MS [24].

#### Serum phospholipid FA concentration measurements

Serum PLFA concentrations were measured using gas chromatography [15], resulting in the 41 PLFA relative concentrations listed in Table 3 of [15]. PLFAs with ≥1 unsaturated double bonds were classified according to whether the bonds were *cis* or *trans*.

**TABLE 1:** Feeding Study dietary intakes (grams per day) for fatty acids and other dietary variables considered for biomarker development in the Women’s Health Initiative (*n* = 153)

Variable^1^	Geometric mean	95% confidence range^2^	Variable	Geometric mean	95% confidence range	Variable	Geometric mean	95% confidence range
SFA			MUFA			Composite FAs		
4:0	0.73	0.18, 1.84	14:1	0.03	0.00, 0.14	SFA Total^3^	25.98	15.06, 46.54
6:0	0.40	0.11, 1.05	16:1	1.09	0.58, 2.15	MUFA Total^3^	30.02	17.61, 51.31
8:0	0.31	0.09, 0.93	18:1	27.69	16.11, 47.81	PUFA Total^3^	16.18	8.71, 29.84
10:0	0.60	0.19, 1.41	20:1	0.31	0.12, 0.67	Omega 3 PUFA^4^	2.20	1.11, 6.65
12:0	0.81	0.23, 3.51	22:1	0.06	0.00, 0.33	Omega 6 PUFA^4^	13.72	7.26, 24.75
14:0	2.42	0.81, 5.62	PUFA			Macronutrients		
16:0	13.45	7.64, 22.60	18:2	13.54	7.17, 24.57	Total fat	79.28	50.96, 128.89
17:0	0.10	0.02, 0.26	18:3	1.85	0.90, 6.37	Total carbohydrates	212.29	129.74, 330.86
18:0	5.87	3.18, 10.27	18:4	0.01	0.00, 0.06	Total protein	77.50	50.12, 112.50
20:0	0.17	0.08, 0.32	20:4	0.11	0.04, 0.22	—	—	—
22:0	0.11	0.03, 0.41	20:5	0.06	0.00, 0.26	—	—	—
—	—	—	22:5	0.03	0.00, 0.08	—	—	—
—	—	—	22:6	0.12	0.01, 0.43	—	—	—

Abbreviations: FA, fatty acid; MUFA, monounsaturated fatty acid; PUFA, polyunsaturated fatty acid; SFA, saturated fatty acid.

1 FA X:Y-X is the number of carbon atoms in the FA molecule; Y is the number of unsaturated double bonds. See Table 2 for common names of the individual FAs.

2 95% confidence range defined as 2.5 to 97.5 percentiles of the distribution.

3 Defined as the sum of the densities for pertinent FAs as listed in Table 1.

4 Also denoted as n–3 and n–6 FAs, respectively.

### Dietary variables considered for biomarker development

Biomarker development was considered for the densities of each of the 11 SFAs, 5 MUFAs, and 7 PUFAs listed in Table 1, as well as for related composite dietary densities. These were the FAs generated by the NDSR food and nutrient database (University of Minnesota, version 2005) for feeding study intakes. Total energy intake (kilocalories per day) from these same sources [13] has a geometric mean (95% confidence interval) of 1904 (1395, 2582). The dietary densities for specific FAs were defined as the ratio (grams per day) divided by total energy intake (kilocalories per day). The composite densities considered were for total SFA, total MUFA, total PUFA, as well as their omega (ω) (n–3) and ω-6 (n–6) components, total protein, and total carbohydrate. The composite FA densities were defined by summing over the relevant specific FA densities in Table 1. The biomarker developments for these composite variables differ from those in our recent publication [12] only through the potential inclusion of measured PLFA concentrations in biomarker equations.

**TABLE 2:** Linear regression CV-*R*^2^ values^1^ for potential biomarker equations for log-transformed fatty acid densities and related composite density variables (*n* = 153)

Density variable	CV-*R*^2^ (%)	Density variable	CV-*R*^2^ (%)	Density variable	CV-*R*^2^ (%)
SFA (common name)		MUFA (common name)		Composite FAs	
4:0 (butyric)	64.7	14:1 (myristoleic)	4.5	SFA total^2^	46.4
6:0 (caproic)	60.9	16:1 (palmitoleic)	21.3	MUFA total^2^	29.9
8:0 (caprylic)	48.7	18:1 (oleic)	31.3	PUFA total^2^	52.8
10:0 (capric)	53.0	20:1 (eicosenoic)	22.8	Omega 3 (n–3) PUFA	46.1
12:0 (lauric)	39.9	22:1 (erucic)	23.4	Omega 6 (n–6) PUFA	52.4
14:0 (myristic)	61.0	PUFA (common name)		Macronutrients	
16:0 (palmitic)	42.2	18:2 (linoleic)	51.7	Total fat	12.4
17:0 (heptadecanoic)	28.4	18:3 (α-linolenic)	50.1	Total carbohydrates	38.4
18:0 (stearic)	34.2	18:3 (γ-linolenic)	24.5	Total protein	37.9
20:0 (arachidic)	34.8	20:4 (arachidonic)	39.7	—	—
22:0 (decosanoic)	49.9	20:5 (eicosapentanoic-EPA)	40.2	—	—
—	—	22:5 (docosapentanoic-DPA)	53.5	—	—
—	—	22:6 (docosohexanoic-DHA)	47.9	—	—

Abbreviations: CV-*R*^2^, cross-validated percent of variation explained;

FA, fatty acid; MUFA, monounsaturated fatty acid; PUFA, polyunsaturated fatty acid; SFA, saturated fatty acid.

1 CV-*R*^2^ values listed here derive from total CV-*R*^2^ values from the linear regression with LASSO-selected variables (maximum 15). Total CV-*R*^2^ is computed from averages of CV-*R*^2^ values from 100 random splits of the data into roughly equal-sized training and validation sets.

2 Defined as the sum of the densities for pertinent FAs as listed in Table 2.

### What about fatty acid supplements?

Since dietary supplement use was self-reported in NPAAS-FS, we focus here on dietary FA intakes, for which intake was provided for biomarker development. However, if a noteworthy fraction of total intake for a FA derives from dietary supplements that influence the concentration of pertinent metabolites, then the ability to develop a strong biomarker for the dietary FA density may be limited. This ability will be studied for all dietary densities considered, including PUFA categories for which supplement use is commonplace. For example, n–3 supplements were used by 41% of the NPAAS-FS cohort at the time of study conduct, mostly (37%) from fish oils. Among users, the average intake of EPA plus DHA was 512 mg/d (SD: 374 mg/d) [25]. A parallel set of biomarker development activities could be considered for the sum of FA intake from the provided diet plus FA intake from supplements (divided by total energy intake). However, the detailed dietary supplement data collected in NPAAS-FS are complex and require considerable curation before being suitable for this purpose (e.g., some ω-3 supplements do not provide dose information). Accordingly, we regard this type of potential diet plus supplement biomarker development as beyond the scope of the present contribution.

### Baseline FA intake in WHI cohorts

As further background on the WHI cohort, from FFQ data at WHI enrollment (1993–1998) SFA intake averaged 19.2 g/d, most of which was either palmitic FA (10.3 g/d) or stearic FA (5.1 g/d); MUFA intake averaged 21.7 g/d, most of which was oleic FA (20.1 g/d); and PUFA intake averaged 11.8 g/d, nearly all of which was either linoleic FA (10.3 g/d) or α-linolenic FA (1.2 g/d).

### Outcome ascertainment and cohort follow-up

Clinical outcomes were reported biannually in the DM trial and annually in the OS, by self-administered questionnaire [16] throughout the time from enrollment in 1993–1998 to the end of the intervention period (31 March, 2005), and annually thereafter in both cohorts. Following the intervention period, WHI participants had the opportunity to enroll for additional follow-up through 30 September, 2010, and subsequently for additional open-ended follow-up that continues today, with >80% of participants agreeing to continue participation on each occasion. Outcome review and adjudication methods have been described previously [26].

### Dietary biomarker criteria and measurement error modeling assumptions

The identification of suitable criteria for dietary biomarker development is itself a research topic. In earlier work [[8], [9], [10], [11]], we used a cross-validated *R*^2^ (CV-*R*^2^) of ≥36% from linear regression of log- feeding study intake on log- metabolite concentrations and other variables as a key developmental criterion. That work also allowed self-reported dietary intake estimates at the time of WHI enrollment (about 10 y before biomarker study) to be considered for inclusion in biomarker models. In this study, we display potential biomarker equations having CV*-R*^*2*^>30% (CV*-R* of ≥0.55), while not allowing any dietary self-report data to be used in biomarker equation development. In addition to a correlational criterion, dietary biomarkers also need to satisfy specificity and sensitivity criteria, which are among a larger set of criteria given by Dragsted et al. [27] for the critical assessment of dietary biomarkers. These criteria in relation to the dietary FA biomarkers having CV-*R*^2^ >30% will be considered in the Discussion. Corresponding to plausible satisfaction of these criteria, we assume a measurement model in which the targeted macronutrient density values from NPAAS-FS equal the corresponding biomarker density value plus independent random noise. This model, along with normality assumptions, implies that essentially unbiased disease association hazard ratio parameter estimates can be obtained by Cox regression of disease risk on log-transformed biomarker density values. See [[28], [29], [30]] for additional discussion and justification for this approach.

### Statistical methods

#### Biomarker development for densities of specific FAs and for composite dietary variables

Biomarker linear regression equations for specific and composite dietary densities used the same approach in NPAAS-FS as did our earlier macronutrient density biomarker developments [[8], [9], [10], [11], [12]]. All dietary densities were log-transformed, as were serum- and urine-based potential predictor variables and the TEE and UN measures. Participant characteristics (without log-transformation) were considered for inclusion in biomarker equations as described below, as in [[8], [9], [10], [11], [12]]. Least absolute shrinkage and selection operator (LASSO) procedures [31] were used for variable selection with a maximum of 15 variables selected, and cross-validation was used to select the tuning parameter and reduce regression parameter overfitting when estimating correlations of potential biomarker densities with corresponding dietary feeding study densities.

The participant characteristics considered for inclusion in biomarker equations were any dietary supplement use (yes compared with no); self-reported race/ethnicity at WHI baseline (categories used at that time were White, African American, Hispanic, and other that included American Indian/Alaska Native and Asian/Pacific Islander); season of FFQ completion (spring, summer, fall, winter); education (high school or general educational development diploma, schooling after high school, college degree or higher, missing); age (y), measured BMI (k/m^2^), and self-reported leisure physical activity (MET h/wk). As in our previous work [[8], [9], [10], [11], [12]], CV*-R*^*2*^ values were calculated as the mean of CV-*R*^2^ values from 100 random splits of the NPAAS-FS dataset into 2 approximately equal-sized subsets, one for biomarker equation estimation and one for estimating regression *R*^2^ values using the test subset equation in the validation subset. The contribution of a specific metabolite in the final model was evaluated using cross-validated type-I partial *R*^2^, which is calculated as the product of the type-I partial *R*^2^ (partial *R*^2^ when variables above that are already included in the model) and the ratio of the total CV-*R*^2^ to the total *R*^2^. The details of estimated (linear) biomarker equations having total CV-*R*^2^ values of ≥30% are provided here.

By macronutrient density biomarker, we mean that arising from a linear regression equation meeting correlational criteria for log-transformed feeding study intake on corresponding log-transformed metabolite concentrations and other selected variables. These macronutrient density biomarkers can be considered for application to other WHI cohorts, such as the Nutrition Biomarker Study and NPAAS cohorts, within which the requisite metabolite profiles and other variables are available. All analyses were performed using R 4.0 (https://www.r-project.org).

### Ethics

The WHI is funded primarily by the National Heart, Lung, and Blood Institute. Participants provided written informed consent for their overall WHI and NPAAS-FS activities. The biomarker data generation in NPAAS-FS was funded by the National Cancer Institute. Related WHI protocols for NPAAS-FS participants were approved by the Institutional Review Boards at the Fred Hutchinson Cancer Center and at each participating clinical center (clinicaltrials.gov identifier: NCT00000611).

## Results

Table 1 shows geometric means and 95% confidence ranges (CRs) (2.5–97.5 percentile) in NPAAS-FS for each of the dietary variables considered for density biomarker development. Note that FAs having high feeding study geometric mean intakes include FA16:0 (palmitic acid), FA18:0 (stearic acid), FA18.1 (oleic acid), FA18:2 (linoleic acid), and FA18.3 (α-linolenic acid). The 95% CRs in Table 2 indicate noteworthy variation in dietary intake densities, ≥3-fold between upper and lower 95% CR values for most of the variables considered, even higher for several of the specific FA densities. Supplementary Table 2 shows corresponding geometric means (95% CRs) for the same density variables using input data from FFQs covering the 3 mo just before the NPAAS-FS feeding period. These suggest an even broader range of dietary intake, presumably due to the noise component of the associated measurement error.

Table 2 shows linear regression CV-*R*^2^ values for each specific FA (along with the FA common name) as well as for the composite dietary variables considered for biomarker development. Equations having CV-*R*^2^ >30% arose for 11 SFA, 1 MUFA, 6 PUFA densities, n–3, and n–6 PUFA, as well as for each of the other composite dietary variables considered, except for total MUFA density. Note that CV- *R*^2^ values are quite substantial for the dietary FA densities for EPA, decosapentaenoic acid (DPA), DHA, and total ω-3 FAs, for which there may be noteworthy supplement intake in NPAAS-FS.

**TABLE 3:** Biomarker equations^1^ for the densities of log-transformed frequently consumed SFAs (*n* = 153)

SFA 16:0 Palmitic acid			
Regression variable^2^^,^^3^	Coeff	*R*^2^	CV-*R*^2^ (%)
(Intercept)	−6.606	—	—
Phospholipid fatty acid (PLFA 16:1n7t) (serum)	0.357	0.260	18.0
Triacylglycerol (TAG 52:4, FA 16:1) (serum)	−0.182	0.193	13.3
Ceramide (CER 22:0) (serum)	0.387	0.062	4.3
N-carbamoyl-beta-alanine (serum)	0.170	0.034	2.4
Allantoin (urine)	−0.055	0.018	1.2
Triacylglycerol (TAG 52:1, FA 18:1) (serum)	0.088	0.008	0.6
Triacylglycerol (TAG 52:5, FA 16:1) (serum)	0.059	<0.001	<0.1
4-hydroxybenzoic acid (serum)	0.040	0.016	1.1
Total energy expenditure	−0.136	0.005	0.3
Diacylglycerol (16:0, 18:1) (serum)	0.071	0.001	0.1
Urinary nitrogen	0.051	0.006	0.4
Triacylglycerol (TAG 54:5, FA 20:2) (serum)	−0.049	0.003	0.2
Phospholipid fatty acid (PLFA 16:1n9c) (serum)	−0.057	0.002	0.2
Phospholipid fatty acid (PLFA 18:1n7c) (serum)	−0.079	0.001	0.1
Triacylglycerol (TAG 54:1, FA 20:0) (serum)	0.037	0.001	0.1
Total	—	0.611	42.2

SFA 18:0 Stearic acid			

Regression variable^2^^,^^3^	Coeff	*R*^2^	CV-*R*^2^ (%)

(Intercept)	−5.883	—	—
Triacylglycerol (TAG 51:1, FA 18:1) (serum)	−0.335	0.218	13.0
Phospholipid fatty acid (PLFA 16:1n7t) (serum)	0.477	0.054	3.2
Phosphatidylethanolamine (PE P18:0, 20:3)∗ (serum)	0.145	0.044	2.6
Triacylglycerol (TAG 52:1, FA 18:1) (serum)	0.223	0.040	2.4
Triacylglycerol (TAG 56:5, FA 20:2) (serum)	−0.141	0.047	2.8
Ceramide (CER 22:0) (serum)	0.527	0.075	4.5
Phosphatidylcholine (PC18:1, 22:6) (serum)	−0.130	0.026	1.6
Triacylglycerol (TAG 52:1, FA 20:0) (serum)	0.091	0.009	0.5
Total energy expenditure	−0.270	0.023	1.4
Phospholipid fatty acid (PLFA 18:1n7c) (serum)	−0.233	0.020	1.2
Phospholipid fatty acid (PLFA 16:1n9c) (serum)	−0.164	0.012	0.7
Diacylglycerol (DAG 16:0, 18:1) (serum) l	0.144	0.004	0.2
Triacylglycerol (TAG 49:1, FA 18:1) (serum)	0.059	<0.001	<0.1
Urinary nitrogen	−0.027	0.001	<0.1
Total	—	0.572	34.2

Total SFA density			

Regression variable^2^^,^^3^	Coeff	R^2^	CV-R^2^ (%)

(Intercept)	−7.107	—	—
Phospholipid fatty acid (PLFA 16:1n7t)	0.450	0.288	20.5
Triacylglycerol (TAG49:1, FA18:1) (serum)	−0.194	0.130	9.3
Cholesterol ester (CE12:0) (serum)	0.076	0.025	1.8
Diacylglycerol (DAG16:0, 18:1) (serum)	0.243	0.069	4.9
Ceramide (CER22:0) (serum)	0.434	0.033	2.3
Triacylglycerol (TAG52:1, FA18:1) (serum)	0.178	0.026	1.9
Triacylglycerol (TAG56:5, FA20:2) (serum)	−0.088	0.017	1.2
N-carbamoyl-beta-alanine (serum)	0.184	0.020	1.4
Phospholipid fatty acid (PLFA16:1n9c) (serum)	−0.148	0.024	1.7
Triacylglycerol (TAG54:1, FA 20:0) (serum)	0.085	0.006	0.4
Sphingomyelin (SM14:0) (serum)	0.123	0.006	0.4
Phospholipid fatty acid (PLFA 18:1n7c) (serum)	−0.144	0.004	0.3
Total energy expenditure	−0.093	0.003	0.2
Cholesterol ester (CE 15:0) (serum)	0.053	0.001	<0.1
Urinary nitrogen	0.001	<0.001	<0.1
Total	—	0.652	46.4

Abbreviations: Coeff, estimated regression coefficient; CV-*R*^2^, cross-validated percent of variation explained; FA, fatty acid; LPC, lysophosphatidylcholine; PC, phosphatidylcholine; *R*^2^, percent of variation explained; SP, sphingomyelin; TAG, triacylglycerol; TEE, total energy expenditure.

1 Biomarker equations with *R*^*2*^ and corresponding cross-validated *R*^*2*^ (CV-*R*^*2*^) values for each variable, using serum and 24-h urine metabolites and established dietary biomarkers, were developed using specimens collected during 2011–2013 in a 153-participant Nutrition and Physical Activity Assessment study-Feeding study (NPAAS-FS) within the Women’s Health Initiative. CV-*R*^2^ values listed here are the cross-validated type-I partial *R*^2^ values, calculated as the product of the type-I partial *R*^2^ (partial *R*^2^ when variables above that are already included in the model) and the ratio of the total CV-*R*^2^ to the total *R*^2^ from the linear regression with LASSO-selected variables where the total CV-*R*^2^ are computed from averages of CV-*R*^2^ values from 100 random splits of the data into roughly equal-sized training and validation components.

2 All metabolite concentrations, as well as total energy expenditure and urinary nitrogen, were log-transformed. Participant characteristics (without log-transformation) were also considered for inclusion in these equations (see Methods). *P <* 0.10 for selection and retention for all regression variables.

3 In CER X:A; FA X:A; DAG X:A, Y:B; PE P X:A, Y:B; PC X:A, Y:B; SM X:A, and CE X:A, X and Y indicate the number of carbon atoms and A and B indicates the number of double bonds in the fatty acid chains. In TAG X:A, X indicates the total number of carbon atoms, and A indicates the total number of double bonds in the 3 fatty acid chains. In PLFA X:AnYc and PLFA X:AnYt, X indicates the number of carbon atoms, A indicates the number of double bonds, Y indicates the position of the double bond from the methyl end, and c and t indicate *cis* and *trans* configuration of the double bond, respectively, in the fatty acid chains.

Table 3 shows dietary biomarker equation details for the log-transformed densities for FAs 16:0 (palmitic acid) and 18:0 (stearic acid), as well as for total SFA density. The palmitic acid density CV-*R*^2^ of 42.2% derives primarily from a positive association with serum *trans* FA16:1 and an inverse association with serum triacylglycerol (TAG) 52:4 that includes FA16:1, along with a positive association with ceramide (CER) 22:0, and also includes weak associations with other TAGs and serum metabolites. Note that the TAG designation gives the total number of carbon atoms for the 3 FAs contained in the triglyceride, along with the corresponding total number of double bonds, while a related second descriptor gives the designation of the first FAs to “fly off” when the TAG is fragmented in the collision cell of the mass spectrometer. The smaller CV-*R*^2^ of 34.2% for stearic acid arises primarily from an inverse association with TAG51:1 that includes FA18:1, a positive association with the same *trans* FA16:1, along with weaker associations with other TAGs, a phosphatidylcholine (PC), and a phosphatidylethanolamine (PE). For total SFA density, the CV-*R*^2^ of 46.4% derives primarily from a positive association with the same *trans* FA16:1, an inverse association with TAG49:1 that includes FA18:1, a positive association with diacylglycerol (DAG)16:0, 18:1, and a positive association with CER22:0.

The regression analyses of Table 3 were repeated while including an FFQ assessment of each dietary density, from FFQs covering the 3-mo period just before NPAAS-FS conduct, among the variables that could be selected in model building. In each case, the CV-*R*^2^ increases by <1% with this addition.

Biomarker equation details for the other SFA densities that also satisfy a 30% CV-*R*^2^ criterion are given in Supplementary Table 3. For each of these, a single serum metabolite association explains a substantial fraction of the dietary FA CV-*R*^2^ value shown in Table 2. These include an inverse association of dietary FA4:0 density with TAG51:1 that includes FA18:1, a positive association of FA6:0 density with cholesterol ester (CE)15:0, a positive association of FA8:0 density with CE12:0, a positive association of FA10:0 density with the same *trans* FA16:1 as for other SFAs, a positive association of FA12:0 density with CE12:0, positive associations of FA14:0 density with both the same *trans* FA16:1 and with DAG.16:0, 18:1, a positive association of FA20:0 density with FA22:0, and an inverse association of FA22:0 density with CER24:1.

Table 4 provides biomarker equation details for log-transformed dietary FA18:1 (oleic) density, along with corresponding details for log-transformed dietary FA densities 18:2 (linoleic), FA18:3 (α-linolenic), and for total PUFA density. The CV-*R*^2^ of 31.3% for oleic acid is mostly explained by positive associations with (serum) TAG52:2 (which includes FA18:1) and with LPE18:1, and inverse associations with CE20:2 and TAG52:3, which includes FA18:0. The larger CV-*R*^2^ of 51.7% for linoleic acid derives primarily from a large inverse association with CE18:0, an inverse association with TAG52:2 (includes FA20:1), and a positive association with hexosylceramide (HCER) 22:0. The CV-*R*^2^ of 50.1% for α-linolenic acid is mostly attributable to positive associations with serum FFA18:3 and cystathionine, and inverse associations with TAG50:2 and with acetyl lysine. Corresponding biomarker equations for the densities for FA 20:4 (arachidonic), 20:5 (EPA), 22:5 (DPA), 22:6 (DHA), and for the composite ω-3 and ω-6 PUFA categories are given in Supplementary Table 4. Dietary arachidonic acid density has positive associations with TAG54:5 (includes FA22:5), methylhistidine, and with certain PEs (PE O18:0, 20:4, and PE P16:0, 20:4). The density for dietary EPA density has a substantial association with PLFA22:6c; that for DPA has positive associations with methylhistidine, CER24:1, and LPE16:0; and for DHA has positive associations with FA22:6c and methylhistidine. The biomarker equation for dietary n–3 PUFA density has an inverse association with TAG50:3, and a positive association with cystathionine, while that for n–6 PUFA density has inverse associations with CE18:0 and TAG52:2 and a positive association with HCER 22:0.

**TABLE 4:** Biomarker equations^1^ for the densities of frequently consumed log-transformed unsaturated fatty acids

MUFA 18:1 oleic acid			
Regression variable^2^^,^^3^	Coeff	*R*^2^	CV-*R*^2^ (%)
(Intercept)	−12.196	—	—
Triacylglycerol (TAG52:2, FA18:1) (serum)	0.354	0.141	7.8
N-acetyl alanine (serum)	0.374	0.053	2.9
Lysophosphatidylethanolamine (LPE 18:1) (serum)	0.214	0.067	3.7
Cholesteryl ester (CE 20:2) (serum)	−0.222	0.185	10.2
Phosphatidylcholine (PC 16:0, 20:2) (serum)	−0.154	0.007	0.4
Triacylglycerol (TAG52:3, FA18:0) (serum)	−0.235	0.065	3.6
Phosphatidylcholine (PC 16:0, 22:5) (serum)	−0.181	0.021	1.1
Phosphatidylethanolamine (PE P18:1, 18:1) (serum)	0.082	0.011	0.6
Triacylglycerol (TAG52:4, FA20:2) (serum)	−0.074	0.007	0.4
Urinary nitrogen	0.064	0.002	0.1
Triacylglycerol (TAG51:4, FA16:1) (serum)	−0.056	0.004	0.2
Triacylglycerol (TAG54:2, FA18:1) (serum)	−0.074	0.002	0.1
Total energy expenditure	−0.106	0.004	0.2
Triacylglycerol (TAG56:7, FA22:4) (serum)	−0.013	<0.001	<0.1
Diacylglycerol (DAG18:1, 18:1) (serum)	−0.008	<0.001	<0.1
Total	—	0.569	31.3

PUFA 18:2 Linoleic acid			

Regression variable^2^^,^^3^	Coeff	*R*^2^	CV-*R*^2^ (%)

(Intercept)	−9.232	—	—
Hexosylceramide (HCER 22:0) (serum)	0.614	0.121	9.6
Cholesterol ester (CE18:0) (serum)	−0.621	0.284	22.5
Total energy expenditure	0.469	0.009	0.7
Triacylglycerol (TAG52.2, FA20:1) (serum)	−0.136	0.143	11.3
Urinary nitrogen	−0.181	0.030	2.4
Lysophosphatidylcholine (LPC 20:2) (serum)	0.091	0.025	2.0
Cholesterol ester (CE15:0) (serum)	−0.174	0.017	1.4
Triacylglycerol (TAG50:2, FA14:1) (serum)	−0.079	0.005	0.4
Cholesterol ester (CE 16:0) (serum)	−0.450	0.014	1.1
Cholesterol ester (CE18:1) (serum)	−0.223	0.003	0.2
Triacylglycerol (TAG50:2, FA16:1) (serum)	−0.046	0.001	0.1
Triacylglycerol (TAG54:6, FA22:5) (serum)	−0.044	0.001	0.1
Diacylglycerol (DAG18:1, 18:2) (serum)	−0.023	<0.001	<0.1
Triacylglycerol (TAG54:7, FA18:3) (serum)	0.005	<0.001	<0.1
Triacylglycerol (TAG52:2, FA16:1) (serum)	−0.008	<0.001	<0.1
Total	—	0.653	51.7

PUFA 18.3 Alpha linolenic acid			

Regression variable^2^^,^^3^	Coeff	*R*^2^	CV-*R*^2^ (%)

(Intercept)	−3.377	—	—
Cystathionine (serum)	0.245	0.154	11.0
Free fatty acid (FFA 18:3)	0.378	0.248	17.7
N6-acetyllysine (serum)	−0.431	0.081	5.7
Triacylglycerol (TAG50:2, FA14:1) (serum)	−0.161	0.101	7.2
Phosphatidylcholine (PC16:0, 20:2) (serum)	−0.291	0.021	1.5
Triacylglycerol (TAG52:4, FA18:3) (serum)	0.382	0.021	1.5
Triacylglycerol (TAG50:3, FA16:1) (serum)	−0.189	0.016	1.1
Cortisol (serum)	−0.137	0.018	1.3
Cholesterol ester (CE 16:0) (serum)	−0.662	0.025	1.8
Cholesterol ester (CE18:1) (serum)	−0.407	0.008	0.5
Triacylglycerol (TAG54:6, FA22:5) (serum)	−0.104	0.004	0.3
Total energy expenditure	0.258	0.003	0.2
Cholesterol ester (CE18:0) (serum)	−0.223	0.002	0.1
Urinary nitrogen	−0.091	0.002	0.2
Triacylglycerol (TAG49:0, FA16:0) (serum)	0.023	<0.001	<0.1
Total	—	0.703	50.1

Abbreviations: Coeff, estimated regression coefficient; CV-*R*^2^, cross-validated percent of variation explained; FA, fatty acid; LPC, lysophosphatidylcholine; PC, phosphatidylcholine; *R*^2^, percent of variation explained; TAG, triacylglycerol; TEE, total energy expenditure.

1 Biomarker equations with *R*^*2*^ and corresponding cross-validated *R*^*2*^ (CV-*R*^*2*^) values for each variable, using serum and 24-hour urine metabolites and established dietary biomarkers, were developed using specimens collected during 2011–2013 in a 153-participant Nutrition and Physical Activity Assessment study-Feeding study (NPAAS-FS) within the Women’s Health Initiative. CV-*R*^2^ values listed here are the cross-validated type-I partial *R*^2^ values, calculated as the product of the type-I partial *R*^2^ (partial *R*^2^ when variables above that are already included in the model) and the ratio of the total CV-*R*^2^ to the total *R*^2^ from the linear regression with LASSO-selected variables where the total CV-*R*^2^ are computed from averages of CV-*R*^2^ values from 100 random splits of the data into roughly equal-sized training and validation components.

2 All metabolite concentrations, as well as total energy expenditure and urinary nitrogen, were log-transformed. Participant characteristics (without log-transformation) were also considered for inclusion in these equations (see Methods). *P <* 0.10 for selection and retention for all regression variables.

3 In FA X:A; LPE X:A; PC X:A, Y:B; PE P X:A, Y:B; DAG X:A, Y:B; HCER X:A; CE X:A; LPC X:A, and FFA X:A; X and Y indicate the number of carbon atoms, and A and B indicate the number of double bonds in the fatty acid chains. In TAG X:A, X indicates the total number of carbon atoms, and A indicates the total number of double bonds in the 3 fatty acid chains.

In contrast, the CV-*R*^2^ values for the dietary densities of (log-transformed) total MUFA, total PUFA, total carbohydrate, and total protein do not differ meaningfully from our previous analyses, which did not have the separately assessed PFLA densities for use in regression model building. Supplementary Table 5 shows biomarker equations from these analyses for dietary total PUFA, total carbohydrate, and total protein densities.

## DISCUSSION

The presentation above shows biomarker equations for 10 SFA,1 MUFA, and 6 PUFA densities having CV*-R*^*2*^ values of ≥30%, as do equations for n–3 and n–6 PUFA densities, total SFA, total PUFA, total carbohydrate, and total protein densities, based primarily on serum and 24-h urine metabolite concentrations. We previously considered [[8], [9], [10], [11], [12], [13], [14]] biomarker equations for composite macronutrient component densities using these same metabolite platforms, but without the inclusion of 41 PFLAs [15]. The PLFA addition led to a larger CV*-R*^*2*^ for total SFA density, namely, 46.4% compared with. 33.8% in [12], but did not materially alter the biomarker equation CV*-R*^*2*^ values for the other composite macronutrient densities. CV*-R*^*2*^ values for specific FA densities tended to be larger than for corresponding composite FA densities, but the differences were not large.

It is also important to consider whether dietary density biomarker equations plausibly satisfy sensitivity, specificity, and other relevant criteria as these relate to the independence of the error term in our measurement error model described above. Dragsted et al. [27] list 8 criteria for dietary biomarkers, namely, plausibility, dose response, time response, robustness, reliability, specimen stability, analytic performance, and inter-laboratory reproducibility. Many of the influential metabolites in the biomarker equations presented here have biologic plausibility. For example, palmitic and stearic FAs are important for cell membrane integrity and various other physiologic roles and have associations with diabetes and cardiovascular disease risk factors [[32], [33], [34], [35]]. Biomarker equations for these densities have a major positive contribution from *trans* FA16:1. *Trans* FA16:1 is not produced by vegetable oil hydrogenation, and it reflects dairy and meat product intake from ruminant animal sources [36]. Also, tissue concentrations of these FAs are under homeostatic control. High intakes can lead to desaturation and elongation into (*cis*) FA16:1 or FA18:1 and to PUFA lipogenesis. Also, high intake of these SFAs may lead to “spill over” from muscle, liver, or adipose tissue into circulation [35], potentially explaining positive associations with ceramides in biomarker equations. In comparison, low dietary intake of these SFAs could lead to de novo lipogenesis (DNL), mainly involving lipid formation from carbohydrate, to restore homeostasis. For example, DNL primarily led to the production of 16:0, 16:1, 18:0, and 18:1 FAs in a Cardiovascular Health Study report [37]. Hence, one can speculate that inverse associations in Table 3 of FA16:0 and 18:0 densities with various TAGs, DAGs, and CEs, which serve as FA storage and transport units, could reflect relatively low intakes in conjunction with DNL. The metabolites just alluded to explain much of the CV*-R*^*2*^ values for palmitic and stearic acid intake densities in Table 3, as is also the case for the total SFA biomarker equation, for which the CV-*R*^2^ value of 46.4% places it among the stronger biomarker correlates of dietary intake in this population. Many of the same classes of metabolites are also included in the proposed SFA density biomarker equations in Supplementary Table 2.

The CV-*R*^2^ value for the oleic acid density biomarker equation is a more modest 31.3% (Table 4). This equation includes positive contributions from TAG52:2, LPE18:1, and N-acetyl alanine, a nonessential amino acid precursor to fatty acid synthesis. It also includes an inverse association with TAG52:4 that includes FA18:0, and an inverse association with CE20:2, a cholesteryl eicosadienic acid [38] that is perhaps related to favorable associations of FA18:1 with serum lipoprotein fractions. The biomarker equations for intake densities for linoleic acid and linolenic acid each have CV*-R*^*2*^ values >50% (Table 4). The high correlation for FA18:2 derives from a positive association with sphingolipid HCER, also possibly related to a high intake and spill over from muscle, liver, or adipose tissue, and substantial inverse associations with CE18:0 formed from cholesterol and FA18:0, and from TAG52:2 that includes FA20:1. These inverse associations may relate to favorable properties of linoleic acid relative to cholesterol accumulation in cells. For dietary α-linolenic acid density, the high CV*-R*^*2*^ value is substantially attributable to a positive association with serum FFA18:3 itself, and a positive association with cystathionine, an amino acid regulator of FA metabolism, along with inverse associations with TAG50:2 that includes FA14:1, and with acetyl lysine that links metabolism to cell signaling [39]. The potential biomarker equations (Supplementary Table 3) for other dietary PUFA densities that have large CV-*R*^2^ values (arachidonic acid, EPA, DHA, and DPA) involve many of the same metabolite categories, and also positive contributions from urinary methylhistidine, a marker of fish and meat intake, and from PLFA22:6c, which is involved in alpha linolenic and linoleic acid metabolism.

Concerning other Dragsted et al. [27] criteria, the large number and variety of metabolites available for possible inclusion in biomarker equations and the quantitative aspect of the linear regression modeling support dose-response/sensitivity properties for the proposed biomarkers. These biomarker developments are based on specimens collected near the end of a 2-wk feeding period. Feeding study dietary intakes that approximate participants’ habitual diets, and the use of specimen types that respond quickly to diet, may support satisfaction of a “time response” criterion. Our habitual-diet feeding study design results in intakes that are typical of those for the WHI study populations, enhancing the “robustness” of study results, and these intakes provide a “reliable” comparator for evaluating potential biomarkers. As previously reported [24], only 18 of >1000 metabolites from WHI specimens, mainly urinary amino acids measured by NMR, displayed significant alteration with storage time, and other quality control properties for the metabolite platforms utilized have been described [14,15,24]. Comparisons of metabolite assessments with those from the Metabolon platform using the same NPAAS-FS specimens are currently underway.

Collectively, these considerations support plausible satisfaction of the criteria by Dragsted et al. [27] for the biomarkers defined by the equations presented here. This, in conjunction with a CV-*R*^2^ >30% (correlation CV*-R* > 0.55) criterion, supports the suitability of these proposed biomarkers for disease association analysis purposes, at least among postmenopausal US females like those studied in WHI.

The dietary FA density biomarkers defined here have some modest dependence on TEE and UN biomarker values, but do not have meaningful dependence on the set of participant characteristics entertained. This suggests potential transferability to populations having somewhat different distributions for the characteristics considered.

Strengths of the study include a substantial feeding study with multiple metabolite platforms, embedded within a well-characterized prospective cohort, for biomarker development. Limitations include somewhat modest correlations for some FAs, a feeding study that could usefully have a greater sample size and longer duration, and the fact that only postmenopausal US females having fairly homogeneous demographic characteristics were studied.

In summary, serum and urine metabolomic profiles lead to biomarkers for specific dietary FA densities, especially for SFAs and PUFAs, having strong correlations with feeding study intakes and are suited for epidemiologic application in US postmenopausal females. The authors aim, in a separate communication, to apply the biomarkers proposed here to study dietary FA densities at WHI baseline for association with chronic disease incidence in larger WHI cohorts in a regression calibration mode. Resulting associations will be compared with those based on baseline FFQs without biomarker calibration.

## Author contributions

The authors’ responsibilities were as follows – RLP, LFT, MLN, and JLW designed the research. RLP, LFT, MLN, JWL, DR, GANG, XS, YH, and CZ conducted the research and drafted the manuscript. All authors participated actively in the critical evaluation of the manuscript and read and approved the final manuscript. RLP had primary responsibility for the final content.

Decisions concerning study design, data collection and analysis, interpretation of the results, the preparation of the manuscript, and the decision to submit the manuscript for publication resided with committees comprised WHI investigators that included National Heart, Lung, and Blood Institute (NHLBI) representatives. The contents of the paper are solely the responsibility of the authors.

## Data availability

Data, codebook, and analytic code used in this report may be accessed in a collaborative mode as described on the Women’s Health Initiative website (www.whi.org).

## Funding

This work was supported by the National Heart, Lung, and Blood Institute, National Institutes of Health, US Department of Health and Human Services (contracts HHSN268201600046C, HHSN268201600001C, HHSN268201600002C, HHSN268201600003C, HHSN268201600004C, and HHSN271201600004C); National Institute for Diabetes and Digestive and Kidney Diseases grant P30DK035816; National Cancer Institute grants R01 CA119171 and P30 CA15704, and NIH instrumentation grant S10 OD021562.

## Conflicts of interest

No potential conflicts of interest were reported by any author. Dr Neuhouser is an Editor for the Journal of Nutrition and is a Member of that Journal’s Editorial Board. Dr Neuhouser played no role in the Journal’s evaluation of the manuscript.
