# Supplementary material for: Biomarkers for dietary fatty acid densities among postmenopausal United States females derived using a habitual-diet human feeding study
Source: Am J Clin Nutr. 2026 Jan 14;123(3):101197. doi: 10.1016/j.ajcnut.2026.101197 (PMC12975380; doi:10.1016/j.ajcnut.2026.101197)
Supplement: multimedia component 1 [file mmc1.docx]

**Supplementary Materials for**

**Biomarkers for dietary fatty acid densities among postmenopausal U.S. females derived using a habitual diet human feeding study**

**by Ross L. Prentice and Colleagues**

**Table of Contents**

**Supplementary Table 1 (baseline characteristics of NPAAS-FS cohort from Lampe et al (13))** Baseline demographic and lifestyle characteristics of the 153 postmenopausal women who participated in the NPAAS-FS^1^

**Supplementary Table 2.** Food frequency questionnaire^1^ (FFQ) dietary intakes (g/d) for fatty acids and other dietary variables considered for biomarker development (n=153)

**Supplementary Table 3.** Biomarker equations for SFAs that are not commonly consumed in NPAAS-FS and have CV-*R*^2^ >30.0% (n=153)

**Supplementary Table 4.** Biomarker equations for other specific PUFAs that satisfy CV-*R*^2^ >30% (n=153)

**Supplementary Table 5.** Biomarker equations for other macronutrient densities that satisfy CV-*R*^2^ >30% (n=153)

**Supplementary Table 1 (baseline characteristics of NPAAS-FS cohort from Lampe et al (13)).** Baseline demographic and lifestyle characteristics of the 153 postmenopausal women who participated in the NPAAS-FS^1^

Variable Value n (%)

Age^2^

60–69 y 10 (7.0)

70–79 y 127 (83.0)

80–85 y 16 (10.0)

Race-ethnicity^3^

White 146 (95.4)

African American 3 (2.0)

Hispanic 2 (1.3)

Other^4^ 2 (1.3)

Height^2^ cm 162 (157 – 166)^5^

Weight^2^ kg 69.0 (60.8 – 76.4)

BMI (kg/m2)^2^

Normal (<25.0) 61 (39.9)

Overweight (25.0–29.9) 60 (39.2)

Obese (30+) 32 (20.9)

Medication use^2,6^

No 14 (9.2)

Yes 120 (78.4)

Missing 19 (12.4)

Dietary supplement use^2,7^

No 23 (15.0)

Yes 130 (85.0)

Current smoking^2^

No 150 (98.0)

Yes 3 (2.0)

Physical activity^2^ (MET-h/wk)

0–5.5 39 (25.5)

5.6–12.25 38 (24.8)

12.3–24.0 39 (25.5)

24+ 37 (24.2)

Season of enrollment^2^

Spring 38 (24.8)

Summer 51 (33.3)

Fall 31 (20.3)

Winter 33 (21.6)

Education^3^

High school or General Educational Development diploma 10 (6.5)

Schooling after high school 16 (10.5)

College degree or higher 126 (82.3)

Missing 1 (0.7)

^1^Abbreviations: MET-h, metabolic equivalent task hour; NPAAS-FS, Nutrition and Physical Activity Assessment Study - Feeding Study

^2^Measured at time of enrollment in the NPAAS-FS

^3^Collected at time of enrollment in the Women’s Health Initiative

^4^“Other” race included 1 American Indian/Alaska Native and 1 Asian/Pacific Islander

^5^Mean; IQR in parentheses (all such values)

^6^Self-reported prescription and over-the-counter medications

^7^Self-reported dietary supplement use from the NPAAS-FS baseline 4-d food record

**Supplementary Table 2.** Food frequency questionnaire^1^ (FFQ) dietary intakes (g/d) for fatty acids and other dietary variables considered for biomarker development (n=153)

| Variable^1^ | Geom. Mean^2^ | 95% confidence range^3^ | Variable | Geom. Mean | 95% confidence range | Variable | Geom. Mean | 95% confidence range |
| --- | --- | --- | --- | --- | --- | --- | --- | --- |
| **SFA** |  |  | **MUFA** |  |  | Composite FAs |  |  |
| 4:0 | 0.64 | 0.17, 2.06 | 14:1 | 0.05 | 0.01, 0.15 | SFA Total^4^ | 23.91 | 9.24, 54.40 |
| 6:0 | 0.31 | 0.07 1.11 | 16:1 | 1.02 | 0.37, 2.65 | MUFA Total^4^ | 25.84 | 11.50, 64.87 |
| 8:0 | 0.23 | 0.06, 0.78 | 18:1 | 23.48 | 10.30, 59.89 | PUFA Total^4^ | 12.73 | 4.69, 28.24 |
| 10:0 | 0.46 | 0.12, 1.51 | 20:1 | 0.23 | 0.09, 0.56 | Omega 3 PUFA^5^ | 1.42 | 0.68, 3.33 |
| 12:0 | 0.59 | 0.20, 1.85 | 22:1 | 0.01 | 0.00, 0.03 | Omega 6 PUFA^5^ | 11.22 | 4.14, 25.04 |
| 14:0 | 2.15 | 0.74, 6.76 | **PUFA** |  |  | Macronutrients |  |  |
| 16:0 | 12.51 | 5.03, 30.05 | 18:2 | 11.09 | 4.08, 24.58 | Total fat | 67.32 | 29.89, 158.18 |
| 17:0 | 0.08 | 0.02, 0.28 | 18:3 | 1.20 | 0.49, 3.03 | Total carbohydrates | 168.38 | 83.40, 314.31 |
| 18:0 | 5.90 | 2.21, 14.53 | 18:4 | 0.00 | 0.00, 0.01 | Total protein | 67.20 | 30.44, 122.75 |
| 20:0 | 0.16 | 0.06, 0.42 | 20:4 | 0.10 | 0.03, 0.25 |  |  |  |
| 22:0 | 0.17 | 0.04, 0.71 | 20:5 | 0.05 | 0.00, 0.16 |  |  |  |
|  |  |  | 22:5 | 0.02 | 0.00, 0.05 |  |  |  |
|  |  |  | 22:6 | 0.10 | 0.02, 0.40 |  |  |  |

**^1^** FFQs cover a 3-month period just prior to NPAAS-FS conduct

**Supplementary Table 3.** Biomarker equations^1^ for SFAs that are not commonly consumed in NPAAS-FS and have CV-*R*^2^ >30.0% (n=153)

**SFA 4:0 (butyric acid)**

| Regression variable^2,3^ | Coeff | *R*^2^ | CV-*R*^2^ |
| --- | --- | --- | --- |
| (Intercept) | -5.792 |  |  |
| Triacylglycerol (TAG51:1, FA18:1) (serum) | -0.766 | 0.496 | 41.4 |
| Triacylglycerol (TAG52:1, FA18:1) (serum) | 0.770 | 0.008 | 0.7 |
| Triacylglycerol (TAG49:1, FA18:1) (serum) | 0.367 | 0.008 | 0.7 |
| Cholesterol ester (CE15:0) (serum) | 0.687 | 0.096 | 8.0 |
| Phospholipid fatty acid (PLFA 16:1n7t) (serum) | 0.673 | 0.068 | 5.7 |
| Diacylglycerol (DAG14:1, 18:1) (serum) | -0.179 | 0.038 | 3.2 |
| Triacylglycerol (TAG52:1, FA 20:0) (serum) | 0.147 | 0.008 | 0.7 |
| Tagatose (urine) | 0.038 | 0.024 | 2.0 |
| Phospholipid fatty acid (PLFA 16:1n9c) (serum) | -0.283 | 0.006 | 0.5 |
| Phosphatidylcholine (PC16:0, 20:3) (serum) | 0.191 | 0.010 | 0.8 |
| Phospholipid fatty acid (PLFA 18:1n8c) (serum) | 0.166 | 0.007 | 0.6 |
| Phospholipid fatty acid (PLFA 18:1n5c) (serum) | 0.076 | 0.002 | 0.2 |
| Total energy expenditure | -0.135 | 0.002 | 0.2 |
| Urinary nitrogen | -0.070 | 0.001 | 0.1 |
| Triacylglycerol (TAG54:6, FA18:1) (serum) | -0.029 | <0.001 | <0.1 |
| Total |  | 0.775 | 64.7 |

**SFA6:0 (caproic acid)**

| Regression variable^2,3^ | Coeff | *R*^2^ | CV-*R*^2^ |
| --- | --- | --- | --- |
| (Intercept) | -13.555 |  |  |
| Cholesterol ester (CE15:0) (serum) | 0.815 | 0.498 | 40.6 |
| Phospholipid fatty acid (PLFA 16:1n7t) (serum) | 0.759 | 0.032 | 2.6 |
| Triacylglycerol (TAG52:1, FA 18:1) (serum) | 0.381 | 0.111 | 9.1 |
| N-formylmethionine (serum) | 0.388 | 0.029 | 2.4 |
| Tagatose (urine) | 0.048 | 0.027 | 2.2 |
| Triacylglycerol (TAG49:1, FA 18:1) (serum) | -0.144 | 0.003 | 0.2 |
| Triacylglycerol (TAG49:0, FA 16:0) (serum) | 0.102 | 0.003 | 0.2 |
| Diacylglycerol (DAG16:0,18:1) (serum) | 0.298 | 0.012 | 1.0 |
| Cytidine (serum) | 0.114 | 0.009 | 0.7 |
| Phosphatidylcholine (PC16:0, 20:3) (serum) | 0.192 | 0.012 | 1.0 |
| Phospholipid fatty acid (PLFA 22:5n6) (serum) | 0.133 | 0.006 | 0.5 |
| Phospholipid fatty acid (PLFA 16:1n9c) (serum) | -0.223 | 0.005 | 0.4 |
| Urinary nitrogen | 0.006 | <0.001 | <0.1 |
| Total energy expenditure | <0.001 | <0.001 | <0.1 |
| Total |  | 0.747 | 60.9 |

**SFA8:0 (caprylic acid)**

| Regression variable^2,3^ | Coeff | *R*^2^ | CV-*R*^2^ |
| --- | --- | --- | --- |
| (Intercept) | -16.505 |  |  |
| Cholesterol ester (CE12:0) (serum) | 0.301 | 0.370 | 26.0 |
| Free fatty acid (FFA18:1) (serum) | 1.350 | 0.038 | 2.7 |
| Sphingomyelin (SM14:0) (serum) | 0.478 | 0.079 | 5.5 |
| Adenosine (serum) | 0.129 | 0.055 | 3.9 |
| Tagatose (urine) | 0.055 | 0.049 | 3.4 |
| Phospholipid fatty acid (PLFA 16:1n9c) (serum) | -0.325 | 0.022 | 1.6 |
| Phospholipid fatty acid (PLFA 16:1n7t) (serum) | 0.260 | 0.020 | 1.4 |
| Body mass index | 0.015 | 0.022 | 1.6 |
| Cholesterol ester (CE 15:0) (serum) | 0.230 | 0.011 | 0.8 |
| Urinary nitrogen | -0.174 | 0.006 | 0.5 |
| Phospholipid fatty acid (PLFA 18:1n8c) (serum) | 0.133 | 0.001 | 0.1 |
| Phosphatidylcholine (PC18:2, 18:2) (serum) | -0.099 | 0.005 | 0.4 |
| Triacylglycerol (TAG52:2, FA 16:0) (serum) | 0.182 | 0.005 | 0.3 |
| Triacylglycerol (TAG56:6, FA 18:3) (serum) | -0.071 | 0.001 | 0.1 |
| Total energy expenditure | 0.150 | 0.010 | 0.1 |
| Total |  | 0.686 | 48.1 |

**SFA10:0 (capric acid)**

| Regression variable^2,3^ | Coeff | *R*^2^ | CV-*R*^2^ |
| --- | --- | --- | --- |
| (Intercept) | -12.533 |  |  |
| Phospholipid fatty acid (PLFA 16:1n7t) (serum) | 0.610 | 0.272 | 21.3 |
| Tagatose (urine) | 0.077 | 0.086 | 6.8 |
| Cholesterol ester (CE12:0) (serum) | 0.149 | 0.170 | 13.3 |
| Triacylglycerol (TAG49:1, FA18:1) (serum) | -0.229 | 0.059 | 4.7 |
| Diacylglycerol (DAG16:0,18:1) (serum) | 0.444 | 0.032 | 2.5 |
| Triacylglycerol (TAG52:2, FA16:0) (serum) | 0.523 | 0.012 | 0.9 |
| Sphingomyelin (SM14:0) | 0.445 | 0.020 | 1.5 |
| Cholesterol ester (CE15:0) (serum) | 0.281 | 0.004 | 0.3 |
| Triacylglycerol (TAG56:6, FA18:3) (serum) | -0.178 | 0.006 | 0.5 |
| Triacylglycerol (TAG 48:1, FA12:0) (serum) | 0.069 | 0.006 | 0.5 |
| Urinary nitrogen | -0.172 | 0.003 | 0.2 |
| Triacylglycerol (TAG52:1, FA18:1) (serum) | -0.140 | <0.001 | <0.1 |
| Total energy expenditure | 0.225 | 0.002 | 0.2 |
| Phosphatidylcholine (PC17:0, 18:1) (serum) | 0.139 | 0.002 | 0.2 |
| Triacylglycerol (TAG54:5, FA 20:2) (serum) | -0.106 | 0.001 | 0.1 |
| Total |  | 0.676 | 53.0 |

**SFA12:0 (lauric acid)**

| Regression variable^2,3^ | Coeff | *R*^2^ | CV-*R*^2^ |
| --- | --- | --- | --- |
| (Intercept) | -12.262 |  |  |
| Cholesterol ester (CE12:0) (serum) | 0.472 | 0.385 | 23.5 |
| Free fatty acid (FFA18:1) (serum) | 1.553 | 0.041 | 2.5 |
| Uridine (serum) | -0.756 | 0.047 | 2.8 |
| Adenosine (serum) | 0.163 | 0.044 | 2.7 |
| Phosphatidylcholine (PC18:2, 20:4) (serum) | -0.403 | 0.038 | 2.3 |
| Sphingomyelin (SM14:0) (serum) | 0.450 | 0.028 | 1.7 |
| Tagatose (urine) | 0.0421 | 0.021 | 1.3 |
| Phospholipid fatty acid (PLFA 16:1n7t) (serum) | 0.242 | 0.009 | 0.5 |
| Exercise times/week | -0.004 | 0.006 | 0.4 |
| Total energy expenditure | 0.395 | 0.008 | 0.5 |
| Sulfate (urine) | 0.037 | 0.011 | 0.7 |
| 3-hydroxybutyric acid (serum) | 0.068 | 0.006 | 0.3 |
| Enrollment (NPAAS-FS) in fall quarter | 0.103 | 0.006 | 0.4 |
| Thyroxine (serum) | 0.231 | 0.004 | 0.2 |
| Urinary nitrogen | 0.013 | <0.001 | <0.1 |
| Total |  | 0.655 | 39.9 |

**SFA14:0 (myristic acid)**

| Regression variable^2,3^ | Coeff | *R*^2^ | | CV-*R*^2^ |
| --- | --- | --- | --- | --- |
| (Intercept) | -9.503 | |  |  |
| Phospholipid fatty acid (PLFA 16:1n7t) (serum) | 0.564 | | 0.359 | 29.0 |
| Diacylglycerol (DAG16:0, 18:1) (serum) | 0.501 | | 0.211 | 17.0 |
| Cholesterol ester (CE12:0) (serum) | 0.135 | | 0.085 | 6.8 |
| Triacylglycerol (TAG49:1, FA18:1) (serum) | -0.177 | | <0.001 | <0.1 |
| Triacylglycerol (TAG52:1, FA18:1) (serum) | 0.340 | | 0.018 | 1.4 |
| Cholesterol ester (CE15:0) (serum) | 0.288 | | 0.011 | 0.9 |
| Phospholipid fatty acid (PLFA 16:1n9c) (serum) | -0.284 | | 0.018 | 1.4 |
| Sphingomyelin (SM14:0) (serum) | 0.284 | | 0.003 | 0.3 |
| Tagatose (urine) | 0.032 | | 0.023 | 1.9 |
| 5’-methylthioadenosine (serum) | 0.223 | | 0.020 | 1.6 |
| Phospholipid fatty acid (PLFA 18:1n8c) (serum) | 0.125 | | 0.004 | 0.3 |
| Phosphatidylcholine (PC16:0, 20:3) (serum) | 0.131 | | 0.005 | 0.4 |
| Total energy expenditure | -0.067 | | <0.001 | <0.1 |
| Urinary nitrogen | 0.010 | | <0.001 | <0.1 |
| Total |  | | 0.755 | 61.0 |

**SFA20:0 (arachidic acid)**

| Regression variable^2,3^ | Coeff | *R*^2^ | CV-*R*^2^ |  |
| --- | --- | --- | --- | --- |
| (Intercept) | -11.867 |  |  |  |
| Phospholipid fatty acid (PLFA 22:0) (serum) | 0.422 | 0.207 | 12.1 |  |
| Ceramide (CER24:1) (serum) | -0.404 | 0.078 | 4.6 |  |
| Indole-3-carboxylic acid (serum) | -0.192 | 0.029 | 1.7 |  |
| Phosphatidylcholine (PC18:0, 22:5) (serum) | -0.270 | 0.077 | 4.5 |  |
| Hexosylceramides (HCER 24:1) (serum) | -0.381 | <0.001 | <0.1 |  |
| Cholesterol ester (CE17:0) (serum) | -0.231 | 0.075 | 4.4 |  |
| N-acetyl alanine (serum) | 0.313 | 0.043 | 2.5 |  |
| Triacylglycerol (TAG54:2, FA18:1) (serum) | 0.226 | 0.040 | 2.3 |  |
| Phosphatidylcholine (PC16:0, 20:2) (serum) | -0.207 | 0.019 | 1.1 |  |
| Triacylglycerol (TAG51:2, FA18:2) (serum) | -0.167 | 0.011 | 0.6 |  |
| Total energy expenditure | -0.220 | 0.005 | 0.3 |  |
| Cysteinyl glycine (serum) | 0.141 | 0.007 | 0.4 |  |
| 5’-methylthioadenosine (serum) | 0.114 | 0.003 | 0.2 |  |
| Hexosylceramides (HCER22:0) (serum) | 0.172 | 0.002 | 0.1 |  |
| Urinary nitrogen | -0.038 | 0.001 | <0.1 |  |
| Total |  | 0.595 | 34.8 |  |
|  |  |  |  |  |

**SFA22:0 (decosanoic acid)**

| Regression variable^2,3^ | Coeff | *R*^2^ | CV-*R*^2^ |
| --- | --- | --- | --- |
| (Intercept) | -3.445 |  |  |
| Ceramide (CER24:1) (serum) | -1.567 | 0.379 | 26.5 |
| Phospholipid fatty acid (PLFA 22:0) (serum) | 1.445 | 0.059 | 4.1 |
| Phospholipid fatty acid (PLFA 23:0) (serum) | -1.528 | 0.132 | 9.2 |
| Phosphatidylcholine (PC18:0,18:1) (serum) | -0.761 | 0.041 | 2.9 |
| Triacylglycerol (TAG52:4, FA16:1) (serum) | 0.467 | 0.028 | 2.0 |
| Triacylglycerol (TAG58:2, FA18:1) (serum) | 0.140 | 0.033 | 2.3 |
| Free fatty acid (FFA14:0) (serum) | -0.549 | 0.015 | 1.0 |
| Triacylglycerol (TAG53:1, FA18:0) (serum) | 0.234 | 0.001 | 0.1 |
| Phospholipid fatty acid (PLFA 22:1n9) (serum) | 0.381 | 0.012 | 0.9 |
| Lysophosphatidylcholine (LPC22:5) (serum) | -0.158 | 0.006 | 0.4 |
| Sphingomyelin (SM24:0) (serum) | 0.601 | 0.006 | 0.4 |
| Total energy expenditure | -0.103 | <0.001 | <0.1 |
| Urinary nitrogen | 0.052 | <0.001 | <0.1 |
| Hexosylceramides (HCER22:0) (serum) | 0.098 | <0.001 | <0.1 |
| Triacylglycerol (TAG51:2, FA18:2) (serum) | -0.014 | <0.001 | <0.1 |
| Total |  | 0.715 | 49.9 |

Abbreviations: Coeff, estimated regression coefficient

^1^Biomarker equations with *R^2^* and corresponding cross-validated *R^2^* (CV-*R^2^*) values for each variable, using serum and 24-hour urine metabolites and established dietary biomarkers, developed using specimens collected during 2011-2013 in a 153-participant Nutrition and Physical Activity Assessment - Feeding Study (NPAAS-FS) within the Women’s Health Initiative. Cross-validated *R^2^* based on average of validation set *R^2^* values from 100 equal-sized random splits of the data set into test and validation components. CV-R^2^ values listed here for specific variables are the cross validated type-I partial *R*^2^ values, calculated as the product of the type-I partial *R*^2^ (partial *R*^2^ when variables above that are already included in the model) and the ratio of the total CV-*R*^2^ to the total *R*^2^ from the linear regression with LASSO selected variables, where the total CV-*R*^2^ is computed from averages of CV-R^2^ values from 100 random splits of the data into roughly equal sized training and validation components.

^2^All metabolite concentrations as well as total energy expenditure and urinary nitrogen were log-transformed. Participant characteristics (without log-transformation) were also considered for inclusion in these equations (see Methods). *P<*0.10 for selection and retention for all regression variables.

^3^In FA X:A; CE X:A; DAG X:A, Y:B; PC X:A, Y:B; FFA X:A ; SM X:A; HCER X:A; PLFA X:A; and CER X:A, X and Y indicate the number of carbon atoms and A and B indicates the number of double bonds in the fatty acid. In TAG X:A, X indicates the total number of carbon atoms and A indicates the total number of double bonds in the three fatty acids. In PLFA X:AnYc, PLFA X:AnYt, and PLFA X:AnY, X indicates the number of carbon atoms, A indicates the number of double bonds, Y indicates the position of the first double bond from the methyl end, and c and t indicate cis and trans configuration of the double bond, respectively, in the fatty acid.

**Supplementary Table 4.** Biomarker equations^1^ for other specific PUFAs that satisfy CV-*R*^2^ >30% (n=153)

**PUFA20:4 (arachidonic acid)**

| Regression variable^2,3^ | Coeff | *R*^2^ | CV-*R*^2^ |
| --- | --- | --- | --- |
| (Intercept) | -5.301 |  |  |
| Triacylglycerol (TAG54:5, FA22:5) (serum) | 0.409 | 0.153 | 9.8 |
| Phosphatidylethanolamine (PE O18:0, 20.4) (serum) | 0.371 | 0.164 | 10.4 |
| 1/3-methylhistidine (serum) | 0.091 | 0.072 | 4.6 |
| Phosphatidylethanolamine (PE P16:0, 20:4) (serum) | 0.290 | 0.080 | 5.1 |
| Ceramide (CER24:1) (serum) | 0.415 | 0.037 | 2.3 |
| Total energy expenditure | -0.507 | 0.026 | 1.7 |
| Triacylglycerol (TAG50:2, FA14:1) (serum) | -0.151 | 0.045 | 2.9 |
| Creatine (urine) | 0.084 | 0.014 | 0.9 |
| Citrulline (serum) | -0.272 | 0.019 | 1.2 |
| Triacylglycerol (TAG54:6, FA16:0) (serum) | -0.129 | 0.002 | 0.2 |
| Phosphatidylethanolamine (PE O16:0, 20:4) (serum) | 0.102 | 0.004 | 0.3 |
| Phosphatidylcholine (PC18:0,18:1) (serum) | -0.184 | 0.004 | 0.3 |
| Phosphatidylcholine (PC18:1,18:3) (serum) | -0.035 | 0.001 | 0.1 |
| Lysophosphatidylcholine (LPC18:3) (serum) | -0.021 | <0.001 | <0.1 |
| Urinary nitrogen | -0.033 | <0.001 | <0.1 |
| Total |  | 0.622 | 39.7 |

**PUFA20:5 (EPA)**

| Regression variable^2,3^ | Coeff | *R*^2^ | CV-*R*^2^ |
| --- | --- | --- | --- |
| (Intercept) | -21.995 |  |  |
| Phospholipid fatty acid (PLFA 22:6n3c) (serum) | 1.730 | 0.332 | 21.0 |
| Triacylglycerol (TAG54:1, FA20:1) (serum) | 0.947 | 0.078 | 4.9 |
| Ceramide (CER24:1) (serum) | 1.493 | 0.047 | 3.0 |
| Cholesterol ester (CE22:4) (serum) | -1.301 | 0.039 | 2.5 |
| Glycochenodeoxycholate (serum) | 0.264 | 0.023 | 1.4 |
| Trimethylamine-N-oxide (serum) | 0.338 | 0.058 | 3.7 |
| Phosphatidylethanolamine (PE18:0,18:0) (serum) | 0.968 | 0.013 | 0.8 |
| 1/3-methylhistidine (serum) | 0.212 | 0.021 | 1.4 |
| Total energy expenditure | -1.23 | 0.010 | 0.6 |
| Trimethylamine-N-oxide (urine) | 0.305 | 0.010 | 0.6 |
| Phosphatidylcholine (PC18:2, 20:4) (serum) | 0.457 | 0.004 | 0.2 |
| Triacylglycerol (TAG56:2, FA20:1) (serum) | 0.179 | 0.001 | 0.1 |
| Urinary nitrogen | 0.149 | 0.001 | <0.1 |
| Triacylglycerol (TAG56.6, FA22:6) (serum) | 0.005 | <0.001 | <0.1 |
| Total |  | 0.637 | 40.2 |

**PUFA22:5 (DPA)**

| Regression variable^2,3^ | Coeff | *R*^2^ | CV-*R*^2^ |
| --- | --- | --- | --- |
| (Intercept) | -19.753 |  |  |
| 1/3-methylhistidine (serum) | 0.234 | 0.201 | 15.4 |
| Ceramide (CER24:1) (serum) | 1.086 | 0.181 | 13.9 |
| Lysophosphatidylethanolamine (LPE16:0) (serum) | 1.059 | 0.124 | 9.5 |
| Phosphatidylethanolamine (PE18:0,18:0) (serum) | 0.685 | 0.065 | 5.0 |
| Phospholipid fatty acid (PLFA 22:6n3) (serum) | 0.441 | 0.015 | 1.2 |
| Triacylglycerol (TAG54:0, FA16:0) (serum) | 0.192 | 0.029 | 2.2 |
| Triacylglycerol (TAG54:1, FA20:1) (serum) | 0.333 | 0.020 | 1.6 |
| Trimethylamine-N-oxide (urine) | 0.179 | 0.030 | 2.3 |
| Triacylglycerol (TAG54:6, FA22:6) (serum) | -0.126 | 0.008 | 0.6 |
| Diacylglycerol (DAG18:0,18:1) (serum) | 0.410 | 0.012 | 0.9 |
| Total energy expenditure | -0.577 | 0.008 | 0.6 |
| Trimethylamine-N-oxide (serum) | 0.097 | 0.002 | 0.2 |
| Triacylglycerol (TAG56:6, FA22:6) (serum) | -0.091 | <0.001 | <0.1 |
| Urinary nitrogen | 0.153 | 0.002 | 0.1 |
| Triacylglycerol (TAG50:4, FA14:1) (serum) | -0.044 | <0.001 | <0.1 |
| Total |  | 0.698 | 53.5 |

**PUFA22:6 (DHA)**

| Regression variable^2,3^ | Coeff | *R*^2^ | CV-*R*^2^ |
| --- | --- | --- | --- |
| (Intercept) | 0.737 |  |  |
| Phospholipid fatty acid (PLFA 22:6n3c) (serum) | 1.035 | 0.358 | 25.9 |
| 1/3-methylhistidine (serum) | 0.267 | 0.095 | 6.9 |
| Urinary nitrogen | 0.901 | 0.008 | 0.6 |
| Triacylglycerol (TAG54:1, FA20:1) | 0.634 | 0.075 | 5.4 |
| Creatinine (urine) | -0.815 | 0.039 | 2.8 |
| Phosphatidylethanolamine (PE18:0,18:0) (serum) | 0.851 | 0.029 | 2.1 |
| Ceramide (CER24:1) (serum) | 0.828 | 0.026 | 1.9 |
| Trimethylamine-N-oxide (serum) | 0.176 | 0.016 | 1.1 |
| Total energy expenditure | -0.660 | 0.008 | 0.6 |
| Acetoacetate (urine) | -0.247 | 0.004 | 0.3 |
| Phosphatidylcholine (PC18:2, 20:4) (serum) | -0.265 | 0.003 | 0.2 |
| Phosphatidylethanolamine (PE P16:0, 22:4) (serum) | -0.135 | 0.001 | 00.1 |
| Triacylglycerol (TAG56:6, FA22:6) (serum) | 0.016 | <0.001 | <0.1 |
| Total |  | 0.661 | 47.9 |

**Omega 3 (n-3) FAs**

| Regression variable^2,3^ | Coeff | *R*^2^ | CV-*R*^2^ |
| --- | --- | --- | --- |
| (Intercept) | -9.090 |  |  |
| Phospholipid fatty acid (PLFA 22:6n3c) (serum) | 1.035 | 0.358 | 25.9 |
| Triacylglycerol (TAG50:3, FA16.1) (serum) | -0.630 | 0.227 | 17.1 |
| Cystathionine (serum) | 0.210 | 0.138 | 10.4 |
| Triacylglycerol (TAG49:2, FA16.1) (serum) | 0.259 | 0.036 | 2.7 |
| Triacyclglycerol (TAG50:2, FA14.1) (serum) | -0.194 | 0.039 | 2.9 |
| Free fatty acid (FFA18:3) | 0.312 | 0.105 | 7.9 |
| Cholesterol ester (CE18:1) (serum) | -0.739 | 0.042 | 3.2 |
| Triacylglycerol (TAG52:0, FA20.0) (serum) | -0.064 | 0.009 | 0.7 |
| Triacylglycerol (TAG52:4, FA18.3) (serum) | 0.230 | 0.004 | 0.3 |
| Total energy expenditure | 0.223 | 0.005 | 0.4 |
| Cholesterol ester (CE:18.0) (serum) | -0.268 | 0.004 | 0.3 |
| Triacylglycerol (TAG49:0, FA16.0) (serum) | -0.046 | 0.001 | 0.1 |
| Cholesterol ester (CE16:1) (serum) | 0.060 | 0.001 | <0.1 |
| Urinary nitrogen | -0.014 | <0.001 | <0.1 |
| Diacylglycerol (DAG16:1,18:1) (serum) | 0.008 | <0.001 | <0.1 |
| Total |  | 0.612 | 46.1 |

**Omega 6 (n-6) FAs**

| Regression variable^2,3^ | Coeff | *R^2^* | CV*-R^2^* |
| --- | --- | --- | --- |
| (Intercept) | -9.11 |  |  |
| Hexosylceramide (HCER22:0) (serum) | 0.600 | 0.118 | 9.5 |
| Cholesterol ester (CE18:0) (serum) | -0.610 | 0.286 | 23.0 |
| Total energy expenditure | 0.456 | 0.009 | 0.7 |
| Triacylglycerol (TAG52:2, FA20:1) (serum) | -0.129 | 0.145 | 11.6 |
| Urinary nitrogen | -0.173 | 0.028 | 2.3 |
| Cholesterol ester (CE15:0) (serum) | -0.177 | 0.018 | 1.4 |
| Lysophosphatidylcholine (LPC20:2) (serum) | 0.088 | 0.024 | 1.9 |
| Triacylglycerol (TAG50:2, FA14:1) (serum) | -0.081 | 0.006 | 0.5 |
| Cholesterol ester (CE:16.0) (serum) | -0.433 | 0.013 | 1.0 |
| Cholesterol ester (CE18:1) (serum) | -0.230 | 0.003 | 0.2 |
| Triacylglycerol (TAG50:2, FA16:1) (serum) | -0.054 | 0.002 | 0.1 |
| Triacylglycerol (TAG54:6, FA22:5) (serum) | -0.038 | 0.001 | 0.1 |
| Diacylglycerol (DAG18:1,18:2) (serum) | -0.028 | <0.001 | <0.1 |
| Triacylglycerol (TAG52:2, FA16:1) (serum) | -0.013 | <0.001 | <0.1 |
| Total |  | 0.652 | 52.4 |

Abbreviations: Coeff, estimated regression coefficient

^1^Biomarker equations with *R^2^* and corresponding cross-validated *R^2^* (CV-*R^2^*) values for each variable, using serum and 24-hour urine metabolites and established dietary biomarkers, developed using specimens collected during 2011-2013 in a 153-participant Nutrition and Physical Activity Assessment - Feeding Study (NPAAS-FS) within the Women’s Health Initiative. Cross-validated *R^2^* based on average of validation set *R^2^* values from 100 equal-sized random splits of the data set into test and validation components. CV-R^2^ values listed here for specific variables are the cross validated type-I partial *R*^2^ values, calculated as the product of the type-I partial *R*^2^ (partial *R*^2^ when variables above that are already included in the model) and the ratio of the total CV-*R*^2^ to the total *R*^2^ from the linear regression with LASSO selected variables, where the total CV-*R*^2^ is computed from averages of CV-R^2^ values from 100 random splits of the data into roughly equal sized training and validation components.

^2^All metabolite concentrations as well as total energy expenditure and urinary nitrogen were log-transformed. Participant characteristics (without log-transformation) were also considered for inclusion in these equations (see Methods). *P<*0.10 for selection and retention for all regression variables.

^3^In FA X:A; PE OX:A, Y:B; PE PX:A, Y:B; CER X:A: PC X:A, Y:B; LPC X:A; CE X:A; PE X:A, Y:B; and DAG X:A, Y:B, X and Y indicate the number of carbon atoms and A and B indicates the number of double bonds in the fatty acid chains. In TAG X:A, X indicates the total number of carbon atoms and A indicates the total number of double bonds in the three fatty acid chains. In PLFA X:AnYc and PLFA X:AnY, X indicates the number of carbon atoms, A indicates the number of double bonds, Y indicates the position of the double bond from the methyl end in the fatty acid chains, and c and t indicate cis and trans configuration of the double bond, respectively, in the fatty acid.

**Supplementary Table 5.** Biomarker equations^1^ for other macronutrient densities that satisfy CV-*R*^2^ >30% (n=153)

**Total PUFA**

| Regression variable^2,3^ | Coeff | *R*^2^ | CV-*R*^2^ |
| --- | --- | --- | --- |
| (Intercept) | -9.029 |  |  |
| Total energy expenditure | 0.423 | <0.001 | <0.1 |
| Hexosylceramides (HCER 22:0) (serum) | 0.445 | 0.088 | 6.8 |
| Cholesterol ester (CE 18:0) (serum) | -0.455 | 0.312 | 24.0 |
| Triacylglycerol (TAG 50:2, FA 14:1) (serum) | -0.107 | 0.115 | 8.8 |
| Urinary nitrogen | -0.168 | 0.021 | 1.6 |
| Cholesterol ester (CE 15:0) (serum) | -0.183 | 0.025 | 1.9 |
| Cholesterol ester (CE 18:1) (serum) | -0.406 | 0.032 | 2.5 |
| Lysophosphatidylcholine (LPC 20:2) (serum) | 0.080 | 0.021 | 1.6 |
| Triacylglycerol (TAG 50:3, FA 16:1) (serum) | -0.136 | 0.035 | 2.7 |
| Cholesterol ester (CE 22:4) (serum) | -0.187 | 0.020 | 1.5 |
| Triacylglycerol (TAG 52:2, FA 20:1) (serum) | -0.062 | 0.001 | 0.6 |
| Free fatty acid (FFA 18:3) (serum) | 0.094 | 0.006 | 0.4 |
| Cholesterol ester (CE 16:0) (serum) | -0.207 | 0.002 | 0.2 |
| Triacylglycerol (TAG 54:6, FA 22:5) (serum) | -0.025 | <0.001 | <0.1 |
| Triacylglycerol (TAG 54:7, FA 18:3) (serum) | 0.009 | <0.001 | <0.1 |
| Total |  | 0.685 | 52.8 |

**Total carbohydrate**

| Regression variable^2,3^ | Coeff | *R*^2^ | CV-*R*^2^ |
| --- | --- | --- | --- |
| (Intercept) | 0.916 |  |  |
| Urinary nitrogen | -0.169 | 0.011 | 0.6 |
| Phospholipid fatty acid (PLFA 16:1n9c) (serum) | 0.200 | 0.105 | 6.2 |
| Triacylglycerol (TAG 52:4, FA 20:2) (serum) | 0.138 | 0.156 | 9.2 |
| Alanine (serum) | -0.206 | 0.077 | 4.5 |
| Phosphatidylcholine (PC 18:0, 22:5) (serum) | 0.155 | 0.122 | 7.2 |
| Total energy expenditure | 0.223 | 0.019 | 1.1 |
| Triacylglycerol (TAG 51:5, FA 18:2) (serum) | 0.072 | 0.033 | 1.9 |
| 4-pyridoxic acid (serum) | -0.025 | 0.036 | 2.1 |
| Maltose (urine) | 0.022 | 0.040 | 2.4 |
| Isovalerylglycine (serum) | -0.029 | 0.014 | 0.8 |
| Triacylglycerol (TAG 50:4, FA 18:2) (serum) | 0.060 | 0.001 | 0.1 |
| Phosphatidylcholine (PC 18:1, 22:5) (serum) | 0.051 | 0.008 | 0.5 |
| Allantoin (urine) | 0.035 | 0.024 | 1.4 |
| Sucrose (urine) | 0.033 | 0.002 | 0.1 |
| Lysophosphatidylcholine (LPC 22:5) (serum) | 0.0267 | 0.003 | 0.2 |
| Total |  | 0.650 | 38.4 |

**Total protein**

| Regression variable^2,3^ | Coeff | *R*^2^ | CV-*R*^2^ |
| --- | --- | --- | --- |
| (Intercept) | -2.393 |  |  |
| Urinary nitrogen | 0.328 | 0.210 | 12.0 |
| 3-hydroxyisovaleric acid (urine) | -0.186 | 0.145 | 8.3 |
| Lysophosphatidylethanolamine (LPE 16:0) (serum) | 0.217 | 0.064 | 3.7 |
| Total energy expenditure | -0.204 | 0.032 | 1.8 |
| Phosphatidylcholine (PC 15:0, 20:4) (serum) | 0.057 | 0.022 | 1.3 |
| Creatine (serum) | 0.069 | 0.082 | 4.7 |
| Methyl glycocholate (urine) | -0.033 | 0.026 | 1.5 |
| Maltose (urine) | -0.021 | 0.028 | 1.6 |
| 2-hydroxybutyrate (serum) | 0.069 | 0.025 | 1.4 |
| Lysophosphatidylcholine (LPC18:1) (serum) | -0.119 | 0.007 | 0.4 |
| 1/3-methylhistidine (serum) | 0.014 | 0.012 | 0.7 |
| Cortisol (serum) | 0.031 | 0.004 | 0.2 |
| 2-oxoisovalerate (serum) | 0.038 | 0.002 | 0.1 |
| Cholesterol ester (CE 22:6) (serum) | 0.017 | 0.001 | <0.1 |
| Propanediol (urine) | -0.005 | <0.001 | <0.1 |
| Total |  | 0.660 | 37.9 |

Abbreviations: Coeff, estimated regression coefficient

^1^Biomarker equations with *R^2^* and corresponding cross-validated *R^2^* (CV-*R^2^*) values for each variable, using serum and 24-hour urine metabolites and established dietary biomarkers, developed using specimens collected during 2011-2013 in a 153-participant Nutrition and Physical Activity Assessment - Feeding Study (NPAAS-FS) within the Women’s Health Initiative. Cross-validated *R^2^* based on average of validation set *R^2^* values from 100 equal-sized random splits of the data set into test and validation components. CV-R^2^ values listed here for specific variables are the cross validated type-I partial *R*^2^ values, calculated as the product of the type-I partial *R*^2^ (partial *R*^2^ when variables above that are already included in the model) and the ratio of the total CV-*R*^2^ to the total *R*^2^ from the linear regression with LASSO selected variables, where the total CV-*R*^2^ is computed from averages of CV-R^2^ values from 100 random splits of the data into roughly equal sized training and validation components.

^2^All metabolite concentrations as well as total energy expenditure and urinary nitrogen were log-transformed. Participant characteristics (without log-transformation) were also considered for inclusion in these equations (see Methods). *P<*0.10 for selection and retention for all regression variables.

^3^In HCER X:A; CE X:A; LPC X:A; FFA X:A; FA X:A; and PC X:A, Y:B, X and Y indicate the number of carbon atoms and A and B indicates the number of double bonds in the fatty acid chains. In TAG X:A, X indicates the total number of carbon atoms and A indicates the total number of double bonds in the three fatty acid chains. In PLFA X:AnYc, X indicates the number of carbon atoms, A indicates the number of double bonds, Y indicates the position of the first double bond from the methyl end, and c indicates cis configuration of the double bond in the fatty acid chains.
